# Artificial Intelligence to Detect Meibomian Gland Dysfunction From *in-vivo* Laser Confocal Microscopy

**DOI:** 10.3389/fmed.2021.774344

**Published:** 2021-11-25

**Authors:** Ye-Ye Zhang, Hui Zhao, Jin-Yan Lin, Shi-Nan Wu, Xi-Wang Liu, Hong-Dan Zhang, Yi Shao, Wei-Feng Yang

**Affiliations:** ^1^Department of Electronic Engineering, School of Science, Hainan University, Haikou, China; ^2^Department of Electronic Engineering, College of Engineering, Shantou University, Shantou, China; ^3^Department of Ophthalmology, Shanghai First People's Hospital, Shanghai Jiao Tong University, National Clinical Research Center for Eye Diseases, Shanghai, China; ^4^Research Center for Advanced Optics and Photoelectronics, Department of Physics, College of Science, Shantou University, Shantou, China; ^5^Jiangxi Centre of National Ophthalmology Clinical Research Center, Department of Ophthalmology, The First Affiliated Hospital of Nanchang University, Nanchang, China; ^6^Department of Mathematics, College of Science, Shantou University, Shantou, China

**Keywords:** deep learning, meibomian gland dysfunction, convolution neural network, *in-vivo* confocal microscopy, DenseNet CNN

## Abstract

**Background:** In recent years, deep learning has been widely used in a variety of ophthalmic diseases. As a common ophthalmic disease, meibomian gland dysfunction (MGD) has a unique phenotype in *in-vivo* laser confocal microscope imaging (VLCMI). The purpose of our study was to investigate a deep learning algorithm to differentiate and classify obstructive MGD (OMGD), atrophic MGD (AMGD) and normal groups.

**Methods:** In this study, a multi-layer deep convolution neural network (CNN) was trained using VLCMI from OMGD, AMGD and healthy subjects as verified by medical experts. The automatic differential diagnosis of OMGD, AMGD and healthy people was tested by comparing its image-based identification of each group with the medical expert diagnosis. The CNN was trained and validated with 4,985 and 1,663 VLCMI images, respectively. By using established enhancement techniques, 1,663 untrained VLCMI images were tested.

**Results:** In this study, we included 2,766 healthy control VLCMIs, 2,744 from OMGD and 2,801 from AMGD. Of the three models, differential diagnostic accuracy of the DenseNet169 CNN was highest at over 97%. The sensitivity and specificity of the DenseNet169 model for OMGD were 88.8 and 95.4%, respectively; and for AMGD 89.4 and 98.4%, respectively.

**Conclusion:** This study described a deep learning algorithm to automatically check and classify VLCMI images of MGD. By optimizing the algorithm, the classifier model displayed excellent accuracy. With further development, this model may become an effective tool for the differential diagnosis of MGD.

## Introduction

The meibomian gland (MG) is a modified secretory sebaceous gland arranged vertically in the upper and lower eyelids and with openings at the eyelid edge (1). The MG can maintain the dynamic balance of the ocular surface by secreting lipids (meibum) into the tear film, thus helping to prevent tear evaporation, to lubricate the eye surface, and form barriers for protection from microbes and other environmental organisms (2–4). Meibomian gland dysfunction (MGD) is a chronic and diffuse meibomian gland disease, the main feature of which is the obstruction of the gland's terminal ducts and/or abnormal meibum secretion (5). The prevalence of MGD ranges from 46.2 to 68.0% in Asians (6). The earliest evaluation of meibomian gland function used the slit lamp and direct observation, but these evaluation methods are approximate. The later infrared imaging technology was effective in diagnosing MGD. It used infrared penetrating camera technology and enhanced contrast function to facilitate meibomian gland imaging (7). It allowed quantitative assessment of the meibomian glands (such as their presence or absence) but not morphological characteristics at the cellular level. In recent years, scholars have found that *in-vivo* laser confocal microscopy imaging (VLCMI) allows observation of the microscopic morphology of the meibomian glands *in-vivo* (8, 9) and investigation of the pathophysiological process of MGD, with particular significance for the diagnosis of MGD.

Artificial intelligence (AI) is a branch of computer science which uses logical operation methods to establish related databases and application models (10). Machine learning is an implementation method of artificial intelligence which extracts generalized rules from data through algorithms characterized by “learning” (11). These rules are represented by mathematical models, including a descriptive analysis of given data. At the same time, other automated methods required experts in the field to define the descriptive rules of the data after which they were implemented by computer programmers in the automated system. The clinical application of AI in ophthalmology diagnosis and treatment includes automatic detection and quantification of ocular lesions or features, automatic screening of ocular diseases, AI-based diagnostic grading, and clinical decision support in retinal treatment and prognostic disease models (12–14). A previous study showed that the application of AI technology in retinal disease was based on the detection of disease-related features on color fundus photography images. The primary retinal markers used in this procedure were large retinal blood vessels and optic discs, sometimes including foveal features, because these markers exist in every fundus image (15). So far, deep learning and other artificial intelligence methods have developed rapidly in ophthalmic research. Glushan's development and validation of deep learning detection algorithms for diabetic retinopathy has been fully recognized (16). Therefore, the development of a deep learning algorithm to automatically identify MGD may reduce the uncertainty of MGD screening and decrease the challenges for human assessors, reducing the need for medical resources and long-term finance.

As far as we know, no diagnostic approach using AI technology for MGD morphological characteristics has been reported previously. Therefore, the purpose of this study was to assess the ability of AI models to detect different types of MGD using VICMIs.

## Methods

### Study Subjects

**Figure 1:**
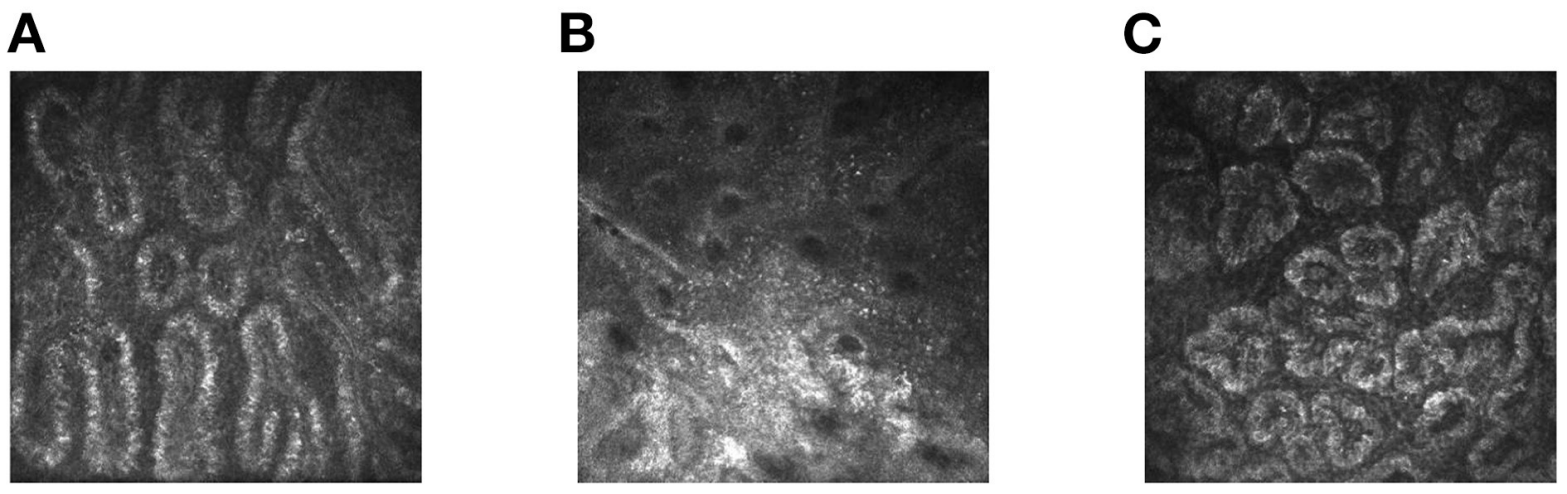
*In-vivo* laser confocal microscope images of obstructive **(A)** and atrophic MGD **(B)** and healthy eyes **(C)**. MGD, meibomian gland dysfunction.

The institutional review board of the First Affiliated Hospital of Nanchang University approved this study, which was conducted in accordance with the tenets of the Declaration of Helsinki. This was a single-center, clinical study. Eight thousand three hundred eleventh VLCMIs were included, among which 2,766 were from healthy controls, 2,744 for diagnosis of OMGD, and 2,801 for diagnosis of AMGD (Figure 1). All the subjects were recruited at the Ophthalmology Department of the First Affiliated Hospital of Nanchang University. Diagnoses were conducted by the ophthalmology expert team and the artificial intelligence screening system. Inclusion criteria for the healthy group were as follows: 1. 18–50 years of age; 2. No significant ocular discomfort and no apparent abnormalities on eyelid margin examination; 3. No eye diseases, no history of eye surgery or trauma and no contact lens wear; 4. No serious systemic disease and able to cooperate to undergo all examinations; 5. Voluntary participation in the study. Inclusion criteria for the MGD group were as follows: 1. Absence of meibomian glands; 2. Abnormal meibomian gland secretion and meibomian gland opening; 3. Changes in quantity and quality of meibomian gland secretions, and any of the above signs combined with symptoms can diagnose MGD. Exclusion criteria: 1. Age younger than 18 or older than 50 years; 2. Patients who are not newly diagnosed in our hospital or have undergone MGD related treatment; 3. Have a history of eye surgery; 4. Have a history of eye trauma; 5. Combined ocular inflammation (such as blepharitis, seborrheic dermatitis, etc.) or eyelid conjunctival scarring disease; 6. Contact lens wearer; 7. Unable to complete all inspections due to other factors; 8. Involuntary take part in this research.

### *In-vivo* Laser Confocal Microscopy

All subjects were examined using the *in-vivo* confocal microscopy (IVCM; Heidelberg Retina Tomograph II-Rostock Cornea Module, Dossenheim, Germany) as previously described (17). Briefly, the subjects were under topical anesthesia, and the eyelid was everted. The center of the Tomo-Cap with comfort gel was placed onto the palpebral conjunctiva and gradually moved from the focal plane into the subconjunctival tissue until the glands were visualized. The images of the underlying meibomian glands were observed and captured by the software (Python 3.7.6; tensorflow GPU 2.0). We scanned the glands while moving the lens from the eyelid margins toward the fornix (vertical movements) and along the palpebral width (horizontal movements). During the examination, no subjects complained of discomfort. The images were two-dimensional with a 400 × 400μm field of view. For each subject, three high-quality digital images of nasal, middle, and temporal glands (total nine images of each eyelid) were selected.

### Definition of Meibomian Gland Abnormalities

Normal meibomian glands were sebaceous glands situated vertically and parallel to each other. Each gland contained grape-like acini, connected by the duct. The acini had convoluted borders with large cells lining and a lumen consisting of fine cellular material (18). Previous study shows that the normal meibomian gland acinar unit density is 113 ± 36.6 glands/mm^2^ (19). In MGD patients, any change in morphology and the number of acinar units may be observed. The diameter of obstructive meibomian acinus was larger than that of normal meibomian acinus, and the acinus contained eyelid fat blockage. Atrophic meibomian acinus was characterized by destruction of acinar epithelial cells with fibrosis (19–21). Based on such changes, we categorized the MGD group images as atrophic or obstructive. The atrophic meibomian glands were fibrosed with abnormal architecture and a reduced acinar unit density. The obstructive glands showed considerable acinar unit enlargement, with the acinar unit density again decreased.

All the subjects underwent evaluation by an ophthalmologist, including tear film breakup time and corneal fluorescein staining, in order to confirm the clinical diagnosis. The VLCMIs were classified by the ophthalmologist into three groups: normal, atrophic, and obstructive.

### Model Construction

**Figure 2:**
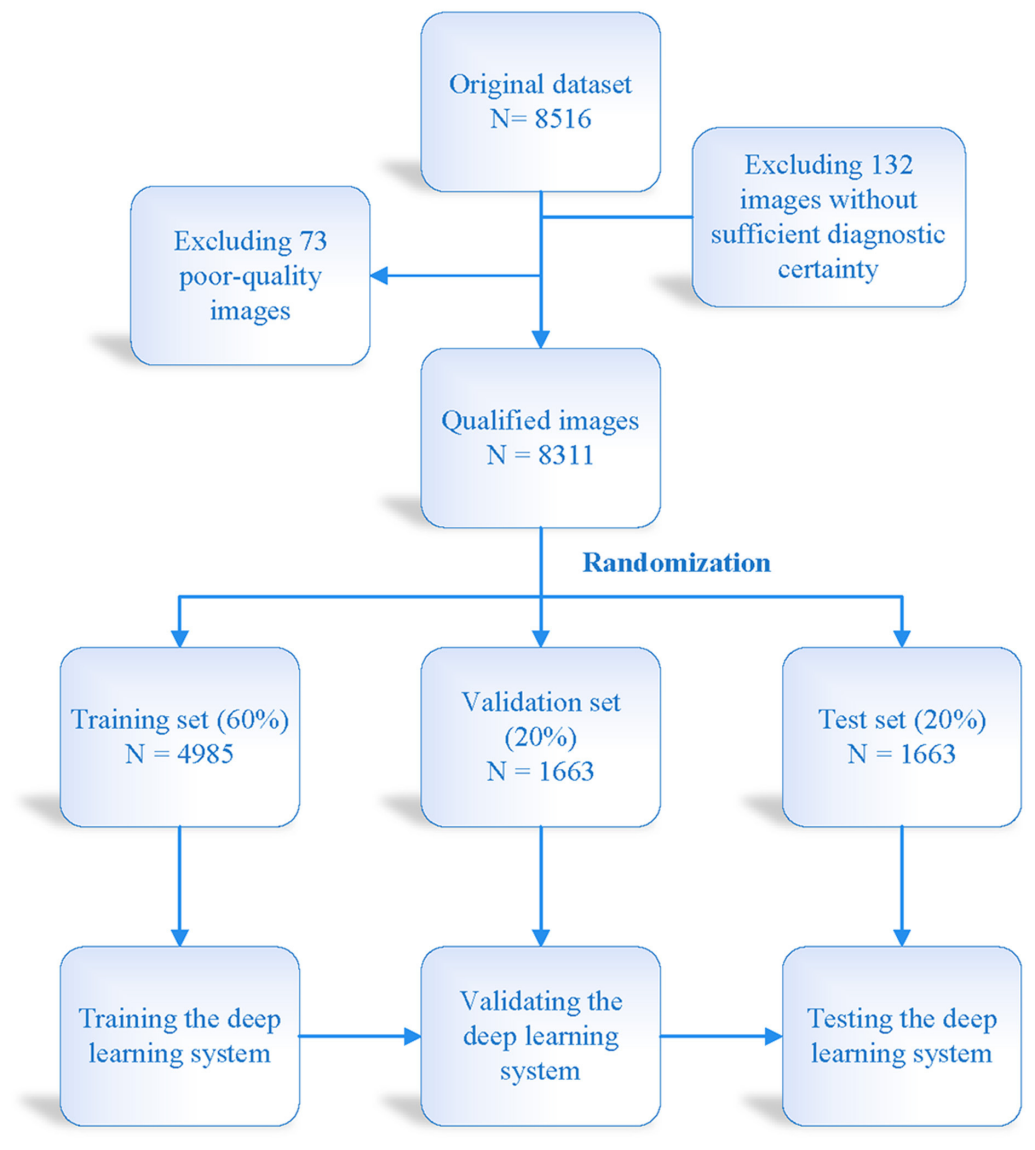
Flow chart illustrating development of the deep learning system.

A total of 4,985 VLCMIs were used for training, and 1,663 VLCMIs were used to test the our deep learning system. Figure 2 shows details of the training data set, the external test data set and the model construction process. We trained three types of network structures (DenseNet 121, DenseNet 169, and DenseNet 201) to classify the meibomian glands into one of the three classes: normal, atrophic, and obstructive. Sparse categorical cross-entropy was performed on the primary data set to differentiate between normal, atrophic, and obstructive. The training started with multiple iterations on a batch size of 16 images, with a learning rate of 0.001, and stopped at 200 epochs. Using the same thresholds, the diagnostic performance of the three classification models was assessed on the five independent external-testing data sets.

### Statistical Analysis

We derived the receiver operating characteristic (ROC) curve by changing the thresholds of ratios of the images classified as different groups and the values output by the neural network. We calculated the area under the ROC curve (AUC). For the sensitivity and specificity, we considered the neural network answer as abnormal if the value output of the ratio of images classified as the two MGD groups was 0.5 or more and standard if the value was <0.5. Finally, we used confusion matrix analysis to evaluate the performance of the automated diagnosis based on the final test results of the three models.

## Results

### Performance of Different Deep Learning Algorithms in the Test Datasets

**Figure 3:**
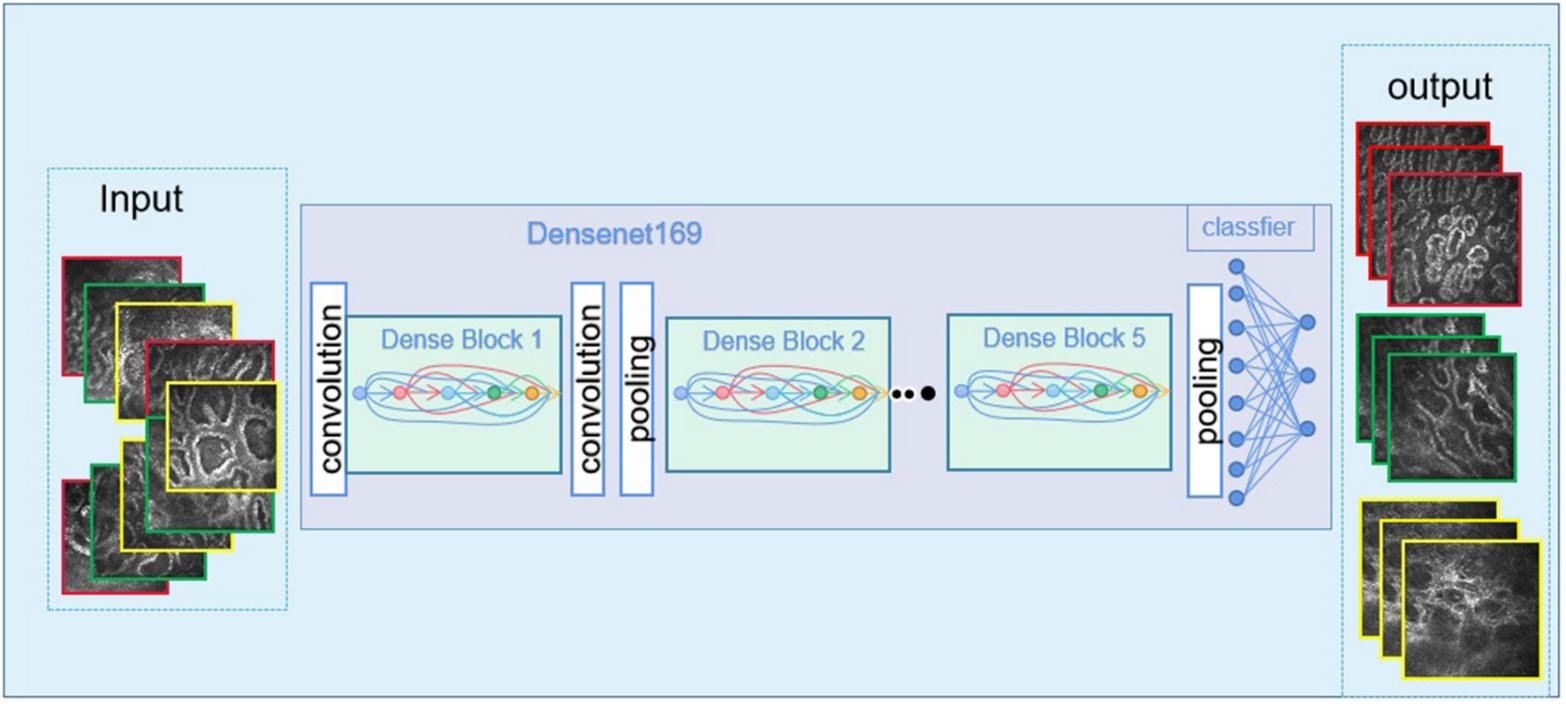
The imaging images go through the deep learning algorithm and the output of the final results.

The ROC curve was used to evaluate the accuracy of the machine model for autonomous recognition of VLCMIs for differential diagnosis between different types of MGD and differentiation between MGD and normal images. Accuracy of the DenseNet 169 model in differential diagnosis of OMGD, AMGD and healthy subjects was 97, 99 and 98%, respectively. The DenseNet 121 model showed accuracy of 94, 96, and 93%, respectively while the DenseNet 201 model showed 94, 97, and 94%, respectively (Figure 3). The DenseNet169 therefore showed highest accuracy. Compared with the correct recognition accuracy of medical experts, the correct recognition accuracy was 91%. The identification accuracy of the DenseNet 169 model was higher than that of the medical experts.

The DenseNet169 model showed sensitivity and specificity of 88.8 and 95.4%, respectively, and AUC of 97.3% for OMGD, 89.4 and 98.4%, respectively and AUC of 98.6% for AMGD, 94.5 and 92.6%, respectively with AUC of 98.0% in normal subjects. Further details and the sensitivities and specificities of the other two DenseNet models are shown in Table 1.

**Table 1:** Performance of three deep learning algorithms in the test dataset.

**Different deep learning models**	**Test dataset**		
	**Sensitivity (95% CI)**	**Specificity (95% CI)**	**Accuracy (95% CI)**
**DenseNet 169**			
Obstructive MGD	88.8% (86.1–91.4%)	95.4% (94.2–96.6%)	97.3% (96.4–98.2%)
Atrophic MGD	89.4% (86.8–91.9%)	98.4% (97.6–99.1%)	98.6% (97.9–99.3%)
Healthy controls	94.5% (92.6–96.4%)	92.6% (91.0–94.1%)	98.0% (97.4–98.6%)
**DenseNet 121**			
Obstructive MGD	85.8% (82.9–88.8%)	87.1% (85.1–89.1%)	93.8% (92.7–95.0%)
Atrophic MGD	69.0% (65.2–72.8%)	99.5% (99.0–99.9%)	95.6% (94.3–96.9%)
Healthy controls	88.8% (86.1–91.4%)	85.2% (83.1–87.3%)	92.7% (91.2–94.1%)
**DenseNet 201**			
Obstructive MGD	89.3% (86.7–91.9%)	85.4% (83.3–87.5%)	94.2% (93.0–95.4%)
Atrophic MGD	70.6% (66.9–74.4%)	99.1% (98.5–99.7%)	96.7% (95.6–97.8%)
Healthy controls	86.4% (83.6–89.3%)	88.6% (86.8–90.5%)	94.1% (92.9–95.3%)

*DenseNet 169 demonstrated the highest accuracy*.

*MGD, meibomian gland dysfunction; CI, confidence interval*.

## Discussion

**Figure 4:**
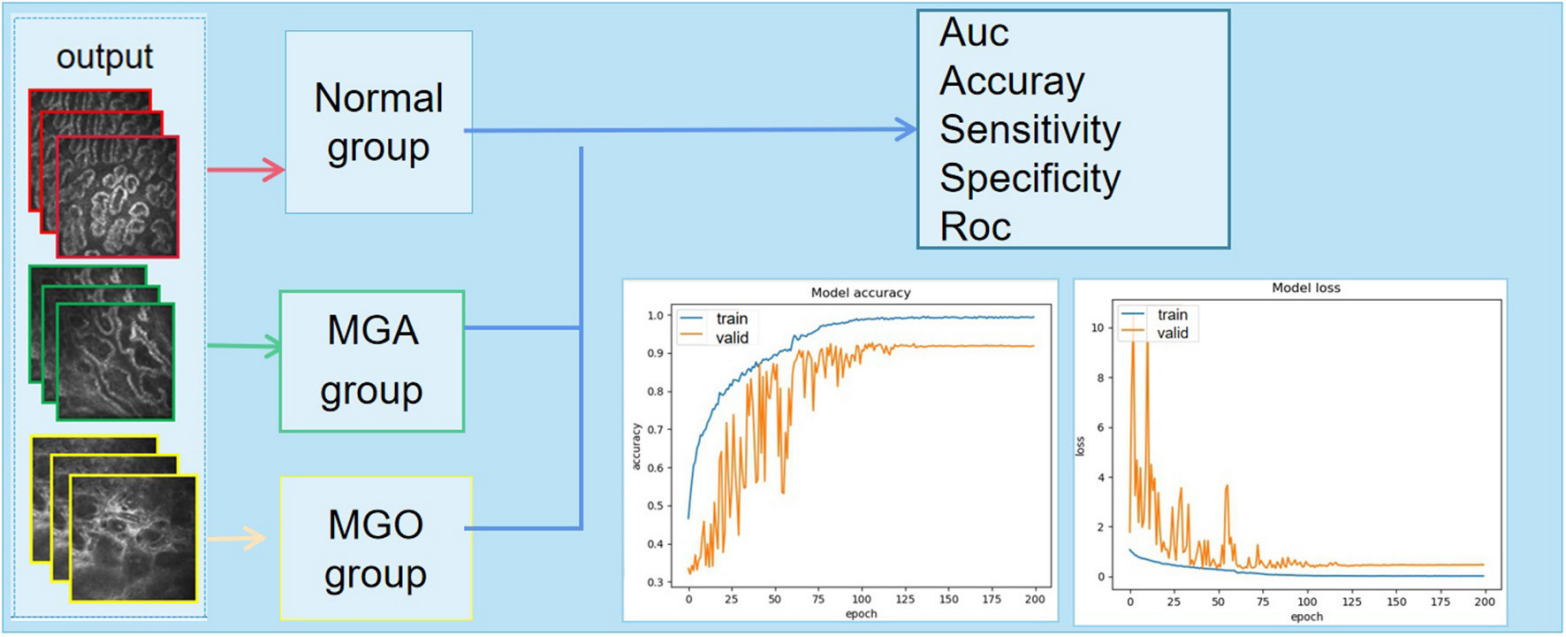
The output of the discriminant result and the final classification accuracy vary with the number of iterations. MGA, atrophic meibomian gland; MGO, obstructive meibomian gland; AUC, area under the curve; ROC, receiver operating characteristic curve.

**Figure 5:**
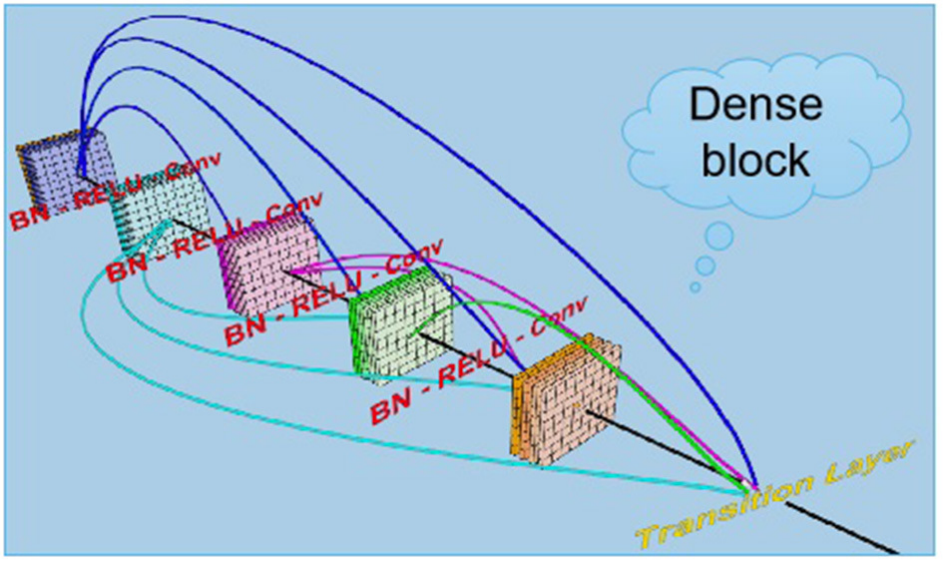
Pooling process of deep learning algorithms.

**Figure 6:**
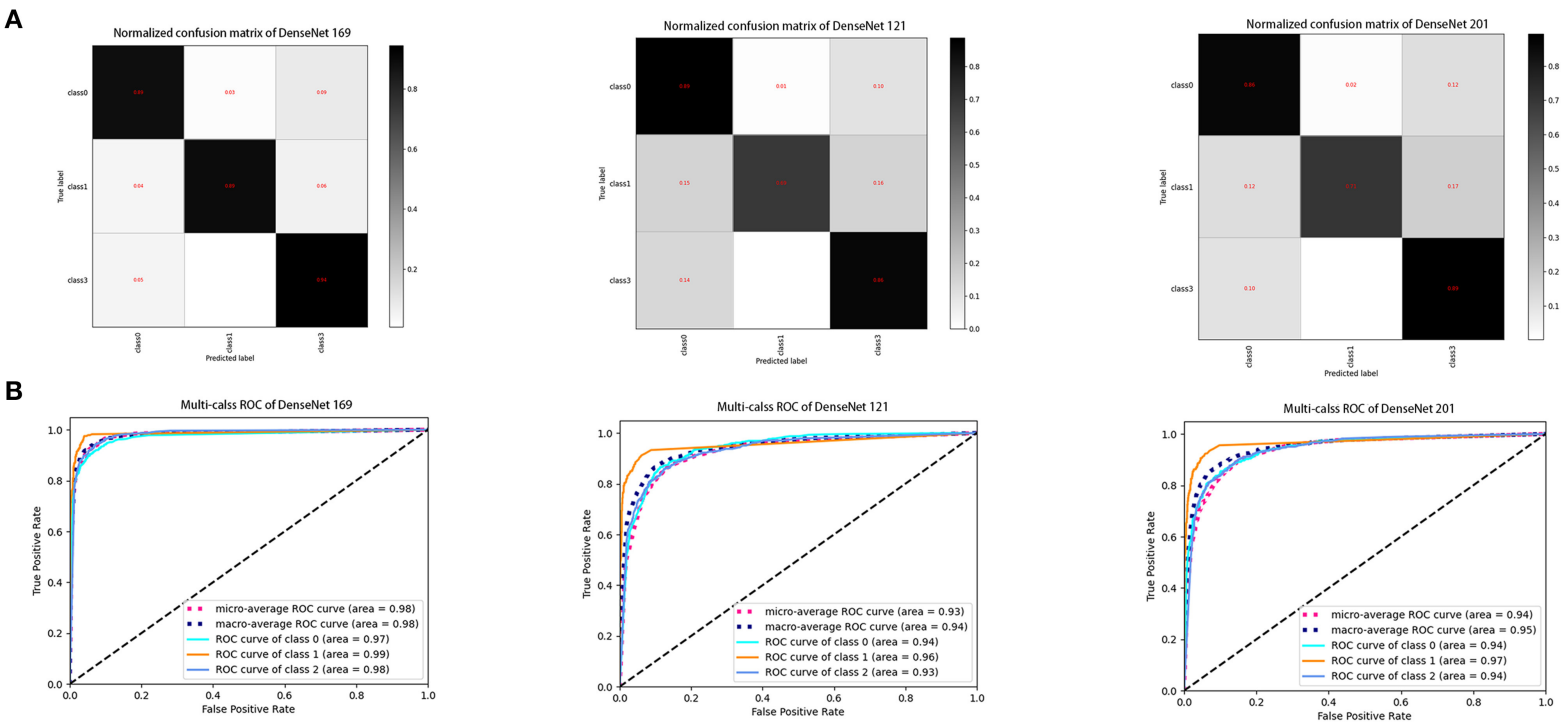
Performance of three DenseNet deep learning algorithms in the test dataset. **(A)** Confusion matrices describing the accuracies of deep learning algorithms. **(B)** Receiver operating characteristic curves indicating the performance of each algorithm in diagnosis of obstructive MGD, atrophic MGD and healthy controls. DenseNet 169 model demonstrated the highest differential diagnostic accuracy. Class 0: obstructive MGD; class 1: atrophic MGD; class 2: healthy controls. MGD, meibomian gland dysfunction.

The aim of the present study was to evaluate performance of the deep learning system to detect different types of MGD in images obtained using an *in-vivo* laser confocal microscope. Our main finding was that the system based on a deep learning neural network can distinguish between OMGD, AMGD and normal subjects (Figures 4, 5), the best performing algorithm being the DenseNet169, with differential diagnostic accuracy of 97 to 99%, sensitivity of over 88% and specificity over 95% (Figure 6).

MGD is a chronic, nonspecific inflammation of the meibomian gland, characterized by duct obstruction or abnormal secretion, and is an important cause of hyperevaporative dry eye (22). In addition, the symptoms of MGD have a significant impact on the quality of life of patients, not only causing eye irritation, but also leading to the sequelae of ocular surface inflammation and visual dysfunction (5). Age is a risk factor for MGD. With increasing age, atrophy of the meibomian gland acinar epithelial cells leads to the large-scale irreversible atrophy of the gland, with a decrease in lipid secretion (23). In recent years, the incidence of MGD in the population has increased, making it the most common disease in ophthalmology clinics. The estimated pooled prevalence of MGD worldwide was 35.8% (24). Consistent with this, a global survey of dry eye patients found that 35% of the population had dry eye, of which 53% were caused by MGD (25). Difficulties in the clinical diagnosis and treatment of MGD included extensive effort required for comprehensive ocular surface analysis and examination, low precision of examination results of MGD-related dry eye, low relevance of the severity of the disease to its management, and poor subjective perception of patient prognosis. At present ophthalmology clinic, the infrared meibography was performed with the Oculus Keratograph 5M^®^ (Wetzlar, Germany) to evaluate the meibomian gland morphology. Images of meibomian glands in the entire lower eyelids were obtained. The meibomian gland function scores were graded according to the extent of the meibomian gland dropout. It was graded from 0 to 3 (0 = no gland loss; 1 = loss <33%, 2 = loss between 33 and 66%, 3 = loss > 66%). This method can also evaluate MGD, but it can only evaluate the morphology of meibomian gland, not meibomian gland acinus (26). Meibomian gland acinus is a cell secreting palpebral fat, which can better represent the function of meibomian gland. However, conventional detection techniques could not directly observe the morphological and quantitative changes of meibomian acinus. These problems restrict the improvement of the diagnosis, treatment level and long-term management of MGD. In addition, due to the low medical and economic resources in underdeveloped and remote areas, the ophthalmologist to patient ratio is low, leading to delayed diagnosis and deterioration of MGD in some patients. For example, in Nigeria, reported physician to patient ratio is as low as 1:2,660 (27). An efficient and socially effective method for evaluating meibomian gland function is therefore needed urgently. The AI-assisted diagnosis of ophthalmic diseases such as cataract, early glaucoma, diabetic retinopathy, and age-related macular degeneration is undergoing rapid development (28–31), but there are few studies on artificial intelligence-assisted diagnosis or screening of dry eye or MGD. We aimed to make a preliminary attempt by studying deep-learning models and conducting a series of in-depth processing of meibomian gland images such as automatic segmentation of meibomian conjunctiva and meibomian glands, morphological feature extraction, and missing rate calculation. The evaluation duration was about 0.5 s, demonstrating sub-second analysis of the meibomian gland images.

To date, the IVCM has been used to qualitatively and quantitatively evaluate the posterior pole, including the optic disc (32). Recently, IVCM has played an increasingly important role in the evaluation of meibomian gland function (17, 33). The advantage of IVCM lies in its imaging of ocular surface tissue *in-vivo* at the cellular level, and its option to observe and measure the microstructure of meibomian glands (34), providing a new perspective from which to understand the pathophysiological mechanism of meibomian gland disease (33). In addition, related studies have further confirmed that IVCM can be used for preclinical diagnosis before the significant loss of glands (18). The arrangement and specific number of acinar units can be observed using the IVCM, and nine confocal images at different angles can be obtained from one side of the eyelid (19). By carefully evaluating the confocal images, we can look for atrophy of acinar units, presence of inflammatory cells, and reduced acinar unit density in MGD patients. The IVCM allows the phenotypic changes in MGD to be described using new diagnostic parameters, such as acinar unit density and acinar unit diameter, reflecting histopathological changes such as glandular atrophy or ductal dilatation, respectively. Healthy control VLCMIs allow the morphological changes of meibomian gland acini to be identified at a preclinical stage, before the appearance of ocular symptoms and morphological changes of the meibomian gland opening. In MGD, there was more neutrophil infiltration into the conjunctiva and some Langerhans cells infiltration into the palpebral conjunctiva, suggesting that the occurrence of MGD was related to conjunctival inflammation. We believed that the micromorphological changes of meibomian glands were earlier than the loss of glands, and the absence of glands appeared ocular symptoms (i.e., MGD) to a certain extent. Therefore, the IVCM may play an important role in the accurate diagnosis of MGD. Combination of the deep learning module developed by our team provides a novel technique for diagnosis of MGD.

The term “DenseNets” reflects its dense connection method, which improves the backpropagation of the gradient, facilitating network training (35). Since each layer can directly reach the final error signal, implicit “deep supervision” is realized. The error signal can rapidly propagate to the earlier layers to obtain direct supervision from the final classification layer. The vanishing gradient is alleviated, and the problem of over-gradient disappearance is more likely to occur at greater network depth (36). This is because the input and gradient information are transferred between many layers, and now this kind of dense connection is equivalent to each layer directly connect the input and loss so that it can reduce the disappearance of the gradient so that the deeper network is not a problem. The number of parameters is reduced, and low-dimensional features are preserved. In a standard convolutional network, the final output will only be used to extract the highest-level features. DenseNet uses high and low feature levels and tends to give a smoother decision boundary, with good performance even with insufficient training data (37). The disadvantage of DenseNet is that due to the need to perform multiple concatenate operations, the data need to be copied multiple times, video memory is rapidly used and dedicated video memory optimization technology is required. In addition, DenseNet is a specialized network, while others such as ResNet are more generic with a broader range of applications. Related deep learning studies in dry eye diseases adopted a VCG19 model, which used machine algorithms to recognize the anterior segment optical coherence tomography images of subjects to independently distinguish dry eye patients from normal subjects, and its recognition sensitivity and specificity reached 86.36 and 82.35%, respectively (38). The advantages of deep learning algorithms in the differential diagnosis of ophthalmic diseases were independent, objective, rapid and non-invasive. With the further development and clinical testing of the model, our model can be used as an important auxiliary means for the diagnosis and screening of meibomian gland dysfunction. From our discussion above, confocal microscopy was very effective in evaluating MGD and dry eye. Artificial intelligence technology can assist in reading pictures, quickly diagnosed and classify them, improve doctors' work efficiency, and help Internet diagnosis and medical treatment in timeliness.

However, there are some limitations in our study. The present model tests the conditions of “meibomian gland atrophy” and “meibomian gland obstruction” only. The meibomian glands may also have structural abnormalities and missing structures, and the model's image recognition accuracy of the meibomian glands still needs to be further improved to detect these in clinical practice. We anticipate collaboration with other hospitals to establish a multi-center database to make data sources more universal and extensive, further improve the accuracy of model interpretation, and promote the further development of artificial intelligence in assisting the diagnosis of ocular surface diseases. In the future, while increasing the sample size, we will also combine other dry eye-related indicators such as the height of the tear meniscus, tear film break up time, and tear secretion to improve the model algorithm further.

In summary, we have found good accuracy of an image depth processing model to evaluate meibomian glands in MGD, and this method can assist clinicians in analyzing the examination results better and faster and provide a more reliable basis for diagnosis. It is of great value as a means to support individualized treatment of dry eye and chronic disease management. This model is most appropriate for general ophthalmology clinics with a low doctor to patient ratio. It can also be used for dry eye screening in the wider population and follow-up and efficacy observation in management of dry eye. Finally, the DenseNet169 model may assist ophthalmologists in the diagnosis of MGD, reducing their workload and pressure related to diagnosis.

## Data Availability Statement

The original contributions presented in the study are included in the article/supplementary materials, further inquiries can be directed to the corresponding author/s.

## Ethics Statement

The studies involving human participants were reviewed and approved by the Medical Ethics Committee of the Shanghai First People's Hospital and First Affiliated Hospital of Nanchang University. The patients/participants provided their written informed consent to participate in this study.

## Author Contributions

Y-YZ, S-NW, and HZ analyze and process data. HZ and J-YL wrote the manuscript. X-WL and H-DZ involved in the data curation. YS and W-FY involved in the study conceptualization, data curation, funding acquisition, and project administration. All authors have read and approved the final manuscript.

## Funding

National Natural Science Foundation (Nos. 82160195 and 91950101); 2020 Li Ka Shing Foundation Cross-Disciplinary Research (No. 2020LKSFG03D); the Optics and Photoelectronics Projects (No. 2018KCXTD011); Central Government Guides Local Science and Technology Development Foundation (No. 20211ZDG02003); Key Research Foundation of Jiangxi Province (Nos. 20181BBG70004 and 20203BBG73059); Excellent Talents Development Project of Jiangxi Province (No. 20192BCBL23020); Natural Science Foundation of Jiangxi Province (No. 20181BAB205034); Grassroots Health Appropriate Technology Spark Promotion Plan Project of Jiangxi Province (No. 20188003).

## Conflict of Interest

The authors declare that the research was conducted in the absence of any commercial or financial relationships that could be construed as a potential conflict of interest.

## Publisher's Note

All claims expressed in this article are solely those of the authors and do not necessarily represent those of their affiliated organizations, or those of the publisher, the editors and the reviewers. Any product that may be evaluated in this article, or claim that may be made by its manufacturer, is not guaranteed or endorsed by the publisher.
